# 1,2-Dibenzoylhydrazine as a Multi-Inhibitor Compound: A Morphological and Docking Study

**DOI:** 10.3390/ijms24021425

**Published:** 2023-01-11

**Authors:** Vincenzo Patamia, Giuseppe Floresta, Chiara Zagni, Venerando Pistarà, Francesco Punzo, Antonio Rescifina

**Affiliations:** Department of Drug and Health Sciences, University of Catania, V.le A. Doria 6, 95125 Catania, Italy

**Keywords:** 1,2-dibenzoylhydrazine, computational drug design, EcR, urease, HIV-1 integrase, crystal morphology, multitarget activity

## Abstract

In the framework of the multitarget inhibitor study, we report an in silico analysis of 1,2-dibenzoylhydrazine (DBH) with respect to three essential receptors such as the ecdysone receptor (EcR), urease, and HIV-integrase. Starting from a crystallographic structural study of accidentally harvested crystals of this compound, we performed docking studies to evaluate the inhibitory capacity of DBH toward three selected targets. A crystal morphology prediction was then performed. The results of our molecular modeling calculations indicate that DBH is an excellent candidate as a ligand to inhibit the activity of EcR receptors and urease. Docking studies also revealed the activity of DBH on the HIV integrase receptor, providing an excellent starting point for developing novel inhibitors using this molecule as a starting lead compound.

## 1. Introduction

**Figure 1:**
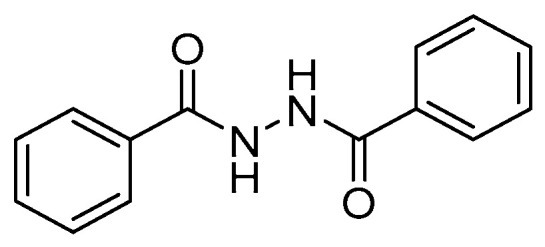
DBH structural formula. Aromatic hydrogens are omitted for the sake of clarity.

The growing evidence of molecules with multitarget properties encourages the shift to joint experimental and computational multitarget approaches [1,2,3,4]. It is now evident that a drug acting on a single receptor is not as effective as expected from the reductionist point of view based on the so-called lock and key model. Recently, our research group has been involved in computational drug design and methodologies to understand structure-activity relationships (SARs), as well as drug repurposing [5,6,7,8,9,10,11,12,13]. Like the lock and key model, one of the current challenges is finding the “master keys” that operate multiple locks to gain access to the desired pharmacological effect. This is a general concept reminiscent of the “molecular master keys” proposed by Müller, who has extensively discussed using privileged structures, frequently employed in medicinal chemistry, to address targets from a family of genes [14]. Another essential aspect being evaluated is the easy accessibility of synthetic potential drugs that can be used for multiple drug targets and would facilitate the work of pharmaceutical industries. In this regard, we have focused on 1,2-dibenzoylhydrazine (DBH) (Figure 1). This compound has been widely used in various pharmacological fields, and finding other targets with different pharmacological applications would increase interest in such compounds and their derivatives [15,16]. Our modeling calculation suggested ecdysone receptor (EcR), urease, and HIV-1 integrase as interesting targets. DBH derivative compounds are non-ecdysteroid activators of insects’ ecdysone receptor complex.

Since these receptors are limited to invertebrates, they represent an attractive target for insecticide development. Indeed, bisacylhydrazine insecticides exert their activity by binding to ecdysone receptors and activating them inappropriately [17]. Their use as insecticides is based on their ability to induce a premature and incomplete molt, during which susceptible insects die from desiccation and starvation [18,19].

Urease is a nickel metalloenzyme that catalyzes the hydrolysis of urea into carbamates and ammonia, thus generating the preferred nitrogen source for many organisms. At the same time, the rise in ammonia levels increases the alkalinity in the stomach, leading to the survival of some bacterial pathogens, such as *Helicobacter pylori*, involved in peptic ulcer disease and stomach cancer [20]. Urolithiasis, urinary catheter encrustation, hepatic coma, hepatic encephalopathy, and pyelonephritis could result from increased urease levels. Compounds containing a fragment of urea or thiourea are a natural choice for the construction of inhibitors of this enzyme [21]. Among these, DBH and its derivatives showed promising results as urease inhibitors [22].

Furthermore, in the context of a human pharmacological application, benzohydrazides have been tested as inhibitors of the human immunodeficiency virus type 1 (HIV-1) integrase [23]. The viral integration process consists of two steps: 3′-processing and strand transfer [24]. Since the active site of the integrase enzymes consists of a motif that coordinates two Mg^2+^ ions [25], the chelation of these metal cofactors represents a promising approach to selective inhibition of strand transfer. A chelating motif and coplanar hydrophobic aryl group are the common pharmacophores for an integrase strand transfer inhibitor [26].

In this framework, for the first time in the literature, we report a molecular modeling study using DBH as a binder on each protein mentioned above. The inhibitory activity of DBH with respect to each enzyme has been compared with some potent ligands reported in the literature, showing, in two cases, better inhibitory activity. Remarkably, DBH has a further application due to its remarkable ability to complex ions such as cobalt, nickel, and cadmium, providing an excellent antimicrobial capacity [27]. The ability to form complexes is due to the absence of substituents linked to nitrogen, and it could be used in different areas, such as heavy metal sequestering or as a sensor. Given the many uses of this compound and its derivatives, after verifying its binding activity, we decided to focus our attention on the structural characteristics of DBH by reporting the crystalline structure, already known, together with the crystal morphologies. This latter information can be of great help while considering all the issues related to active pharmaceutical ingredients (APIs), i.e., their solubilization, dissolution rate, and mechanical properties, which affect the final tableting. In fact, the solid form of pharmaceuticals has a tremendous impact on their material properties. Solubility is one of the main challenges in drug development, and it is particularly relevant in the case of oral medicine. Poor solubility of a pharmaceutical form can cause failure to bring it to market, as happened already in the case of ritonavir [28]. Consequently, the design and study of solid forms in drug products have become a significant research area, particularly when formulation approaches can overcome poor solubility/permeability [29].

## 2. Results and Discussion

### 2.1. Molecular Modeling Studies

The identification of the three targeted proteins was initially conducted using DBH, dibenzoylhydrazine, and hydrazine derivatives as keywords on Scopus and Google Scholar (accessed on 10 September 22). By comparison of the common results from the databases, we selected the most studied targets for developing ligands with a hydrazine moiety. To assess the molecular similarity within the compounds retrieved from the literature and DBH, a pairwise similarity was calculated by using ECFP4/FCFP4 and ECFP6/FCFP6 circular fingerprints. Extended-Connectivity Fingerprints (ECFPs and FCFPs) are circular topological fingerprints optimized and designed for molecular characterization, similarity searching, and ligand-based molecular modeling. They are among the most common similarity search tools in drug discovery and are actually used in many applications. Comprehensive studies demonstrated that these methods are typically among the best-performing search tools [30,31].

**Figure 2:**
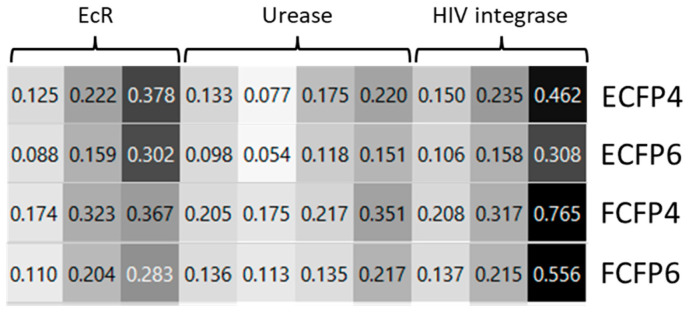
Calculated value (0 low similarity, 1 max similarity) of similarity index between DBH and the most potent compounds acting as ligands of EcR, urease, and HIV-1 integrase with ECFP4/6 and FCFP4/6 fingerprint similarity matrices.

The results of the fingerprint analyses are reported in Figure 2. Circular fingerprints showed that DBH has a structure similar to that of the most potent compounds acting as ligands for EcR, urease, and HIV-1 integrase recovered from the literature [32,33,34,35,36,37,38,39,40]. A docking and MD study was performed to better understand DBH’s chemical interaction with the selected proteins.

The AutoDock Vina software was chosen based on its ability to excellently reproduce both the pose and the binding constant for the co-crystallized ligands in each of the Protein Data Bank (PDB) structures used in this work. To improve the quality of results, after the DBH docking, we performed a 100 ns molecular dynamics (MD) simulation, using the ligand/protein complex in the best-docked pose to ensure the best accommodation of the ligand in the enzyme pocket. Finally, we redocked the ligand using the last 3 ns averaged structure. The workflow of the procedure we used is reported in Figure S1.

We first performed docking studies on the hemipteran EcR (PDB ID: 1Z5X) to evaluate the agonist activity of DBH. Table 1 shows the predicted free energies of binding (Δ*G*) and inhibition constant (*K*_i_) values compared to the co-crystallized Ponasterone A.

**Figure 3:**
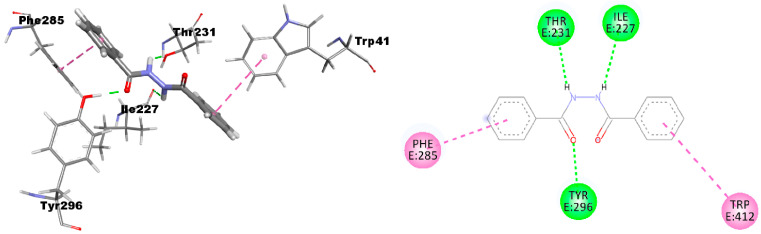
3D and 2D pictures of the DBH interactions within the EcR receptor.

A recent work by Purohit et al. confirms this data, which likewise uses RH-5849, possessing a hydrazine moiety, as a model ligand for docking studies [41]. Looking at the poses within the receptor site (Figure 3), we note that DBH establishes three hydrogen bonds with the residues Ile227, Thr231, and Tyr296 and two *π*-*π* interactions with Phe285 and Trp412 residues. For comparison, the 2D interactions of the reference compound Ponasterone A are reported in Figure S2.

The results obtained from the docking on the urease receptor showed a superior inhibitory activity of DBH compared to thiourea (Table 2), which has often been used in the literature as a standard urease inhibitor [42,43].

**Figure 4:**
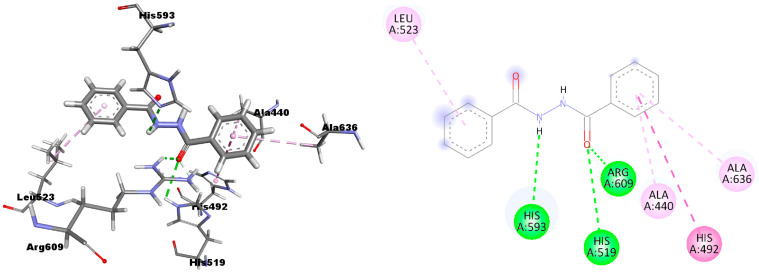
3D and 2D pictures of the DBH interactions within the urease receptor.

Even in this case, the 3D and 2D poses of DBH in the urease receptor site (Figure 4) has three hydrogen bonds with the His519, His593, and Arg609 residues, one *π*-*π* interaction with His492, and three *π*-alkyl interactions with Ala 440, Leu523, and Ala 636. For comparison, the 2D interactions of the reference compound, thiourea, are reported in Figure S3.

The inhibitory activity of DBH against the HIV-1 integrase was compared with that of the compound CHEMBL3259898 (also noted as 4BI or GS-C), currently one of the best inhibitors for this receptor [44]. Although DBH turned out to be less active than CHEMBL3259898 (Table 3), these docking studies lay the foundation to evaluate structural changes to be implemented on DBH to increase the inhibitory activity against the HIV-1 integrase receptor.

**Figure 5:**
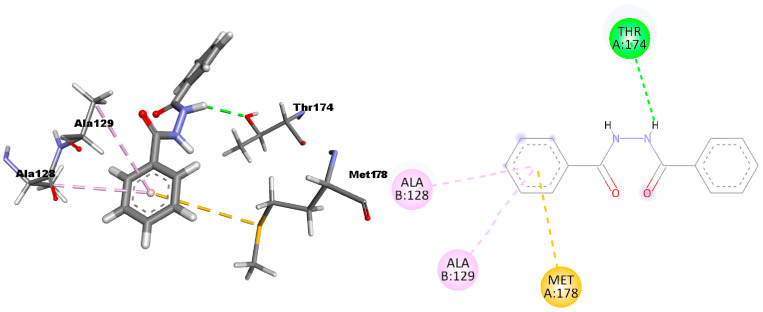
3D and 2D pictures of the DBH interactions within the HIV integrase receptor.

3D and 2D poses at the HIV-1 integrase receptor site show that DBH (Figure 5) establishes one hydrogen bond with the Thr174 residue, two *π*-alkyl interactions with Ala128 and Ala129 residues, and one *π*-sulfur interaction with the Met178. The insertion of some substituents in the DBH’s phenyl groups to increase the interactions with the CHEMBL3259898 compound could be considered. For comparison, the 2D interactions of the reference compound CHEMBL3259898 are reported in Figure S4.

### 2.2. Crystal Morphology Prediction

We used the already-deposited WOBNAC.cif file (the Cambridge Structural Database) to start our calculations. This file is a plain text file used to represent crystallographic information. The compound crystallizes in the monoclinic C 2/c group, and its main cell parameters are *a* = 14.5439(2) Å, *b* = 9.7314(3) Å, *c* = 9.0181(3) Å, and b = 110.9380(10)°.

As a whole, the presence of H-bonds and several non-covalent interactions, or, in other words, of a significant overall bond anisotropy, makes the DBH a challenging case for crystal morphology prediction [46,47].

**Figure 6:**
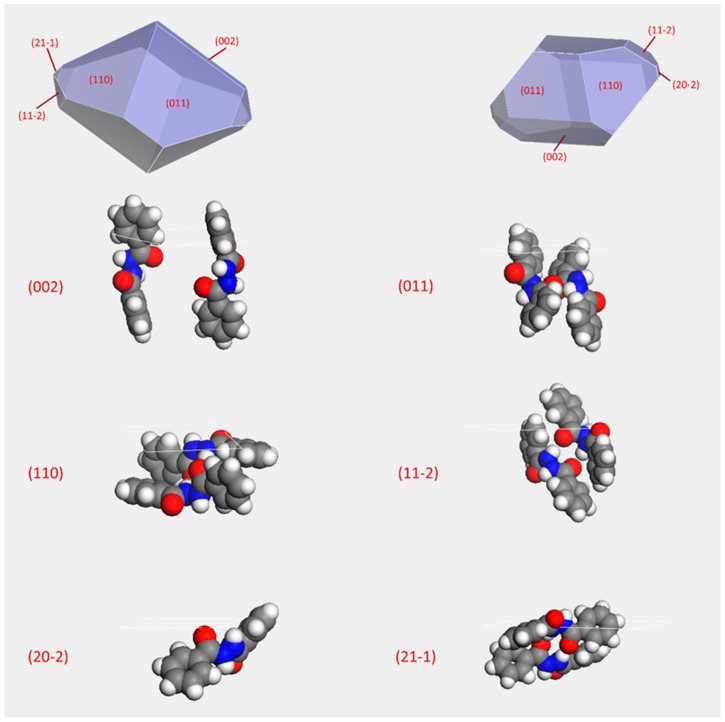
BDH crystal morphology prediction employing the GM (**left**) and BFDH (**right**) methods, respectively. MI faces are reported in round brackets. (002) is missing in the GM model (Table 4) and present in the BFDH prediction only.

Figure 6 reports the predicted morphology obtained by the growth morphology (GM) and Bravais-Friedel-Donnay-Harker (BFDH) methods and the Miller indices of the morphologically important (MI) faces. Table 4 reports the more relevant morphological data.

Although we don’t have any images of the experimental crystal morphology, we can rely on the habit comments left in their CSD deposited file: block-shaped. The predicted morphology and the most protruding groups relative to its MI (Table 4) are sketched in Figure 6.

Both prediction methods could be considered to be in good agreement with the reported blocking habit. However, we should remember that the BFDH method relies on only geometrical considerations and therefore does not account for any energy interaction between the growing crystal faces. We start our analysis by comparing the differently predicted GM and BFDH models.

Figure 6 also sketches the most protruding groups corresponding to the slabs associated with each MI face. Without assuming a specific crystal nucleation model—classical or two-step ones [48]—the presence of a fairly high packing index (69.3% [49]) suggests the lack of residual solvent accessible, and we can assume that the attachment of the solvent took place by the interaction of solvent molecules directly upon the growing crystal face. This is true both for the nucleation, its “inverse” mechanism, and the solubilization. Consequently, we can assume that the actual molecular layout on each crystal face can affect the way different solvents, with different polarity and chemical characteristics, may interact with each considered face. This is why a crystal morphology analysis is a key piece of information for interpreting the solubilization behavior of a given crystalline compound. Therefore, sketching their corresponding slabs, as reported in Figure 6, allows for drawing hypotheses on the possible interactions of the solvent molecules and the chemical environment on the face surface.

Ethanol shows a marked polarity among the most commonly used solvents [50]. A strong interaction between the solvent and the face surface allows a slow, complete, and more extensive crystal face development. In fact, the slower the crystal face grows, the better [51,52]. Only (110) face, predicted in both the GM and BFDH models, although with a different relative weight, shows a very small polar area. There is a constant apolar environment for each MI considered. Therefore, the solvent’s overall effect is almost equal in enhancing or hindering every crystal face growth.

(002) face is a minor MI face basing the calculation on the BFDH method, and it is completely missing when considering the attaching energies. This behavior suggests that this face is energetically penalized. On the other hand, energetics accounts (11–2) as another minor MI face, but it is missing in the BFDH prediction. (110) and (200) show a markedly apolar environment. The use of ethanol may eventually hinder the development of these faces. Conversely, ethanol can enhance the growth of the other MI faces. As a whole, the crystal habit should recall the block shape that is reported in the WOBNAC.cif file. In fact, for the well-known Steno’s law—i.e., the law of constancy of interfacial angles—the angles between two corresponding faces of a crystal are constant and are characteristic of the species; however, as a consequence of several experimental factors, the size of each face may vary, leaving unaltered the relative angles but affecting the crystal habit.

## 3. Materials and Methods

### 3.1. Structure Preparation and Minimization

All the molecules used in this study were built using Marvin Sketch (18.24, ChemAxon Ltd., Budapest, Hungary). The PM6-D3H4 Hamiltonian, implemented in the MOPAC package (MOPAC2016 v. 18.151, Stewart Computational Chemistry, Colorado Springs, CO, USA), was then used further to optimize the 3D structures before the docking calculations. The pairwise similarity was calculated using ECFP4/FCFP4 circular fingerprints using Flare v 6.1 (Cresset Biomolecular Discovery Ltd., Litlington, Cambridgeshire, UK) [31].

### 3.2. Docking and Molecular Dynamics Studies

Flexible ligand docking experiments were performed employing AutoDock Vina implemented in YASARA (v. 22.9.24, Vienna, Austria), using the EcR crystal structure of *Bemisia tabaci* (PDB ID: 1Z5X), the urease crystal structure of *Canavalia ensiformis* (PDB ID: 4H9M), and the HIV-1 integrase crystal structure (PDB ID: 4NYF) retrieved from the PDB_REDO Data Bank. A periodic simulation cell with boundaries extending 5 Å [53] from the surface of the ligand was employed.

The MD simulations of the complexes were performed with the YASARA structure package. A periodic simulation cell with boundaries extending 10 Å [53] from the surface of the complex was employed. The box was filled with water, with a maximum sum of all water bumps of 1.0 Å and a density of 0.997 g/mL.

The setup included optimizing the hydrogen bonding network [54] to increase the solute stability and a p*K*_a_ prediction to fine-tune the protonation states of protein residues at the chosen pH of 7.4 [55,56]. NaCl ions were added at a physiological concentration of 0.9%, with an excess of either Na or Cl to neutralize the cell. Water molecules were deleted to readjust the solvent density to 0.997 g/mL. The final system dimensions were approximately 81 × 81 × 81 Å^3^ for EcR, 101 × 101 × 101 Å^3^ for urease, and 76 × 76 × 76 Å^3^ for HIV integrase protein/ligand complexes.

The simulation was run using the ff14SB force field [57] for the solute, the GAFF2 force field [58] with AM1BCC [59] calculated charges for ligands, and the TIP3P force field for water. The cutoff was 10 Å for van der Waals forces (the default used by AMBER) [60], and no cutoff was applied to electrostatic forces (using the Particle Mesh Ewald algorithm) [61]. The equations of motion were integrated with multiple time steps of 2.5 fs for bonded interactions and 5.0 fs for nonbonded interactions at a temperature of 298 K and a pressure of 1 atm (NPT ensemble) using algorithms described in detail previously [62,63]. A short MD simulation was run on the solvent only to remove clashes. The entire system was then energy minimized using the steepest descent minimization to remove conformational stress, followed by a simulated annealing minimization until convergence (<0.01 kcal/mol Å). Finally, 100 ns MD simulations without any restrictions were conducted, and the conformations of each system were recorded every 200 ps. The last 3 ns averaged structures were considered for redocking.

### 3.3. Crystal Morphology Prediction

The crystal morphology prediction was accomplished through an equilibration protocol based on the Forcite module included in the BIOVIA Material Studio 2021 (Vélizy-Villacoublay, France). For this purpose, molecular mechanics approximation and the Compass III Force Field (FF) were employed [64,65]. The morphology protocol is based on the so-called GM [66,67,68,69,70] and BFDH [71] methods. The calculations were performed in fine detail, allowing a minimum interplanar distance (dhkl) of 1.000 Å without setting any limit to the values of the three MI or the overall number of growing faces.

The morphology prediction accounts for two main contributions to the crystallization energy (*E_cr_*): the energy of each slice (*E_slice_*), i.e., the energy resulting from the lateral interaction of each formula unit within a slice; and the attachment energy (*E_att_*), related to the energy released as a result of the vertical interaction of the formula unit with an underlying slice [72]. This can be summarized as:

(1) $$E_{cr}=E_{slice}+E_{att}$$

It was also demonstrated [69] that the growth rate (*G_r_*) is directly proportional to the attachment energy:

(2) $$G_{r}\propto E_{att}$$

Assuming *E_cr_* to be a constant, an interesting consequence of Equations (1) and (2) is that the bigger the *E_slice_*, the smaller the *E_att_*, and consequently, the slower the growth rate *G_r_*. A relationship between the molecular interactions—intra- and inter-molecular ones—and the crystal morphology can be inferred from the combined analysis of the periodic boundary conditions present in a slice and the *E_att_* related to them. Within this framework, the GM method calculates *E_att_* as stated in eq. 1. For a detailed description of the method, see references [66,67,69,73,74,75,76,77]. All these calculations were carried out at 0 K without surface relaxation. Furthermore, the surface is considered a perfect termination of the bulk.

The search for possible solvent-accessible voids was performed using the VOID algorithm [78], by setting a grid of 0.20 Å and a probe radius of 1.20 Å.

## 4. Conclusions

Interested in the multitarget properties of DBH, we conducted a molecular modeling study of the DBH activity against three receptors to be exploited as potential targets for different pharmacological treatments. The three targeted proteins were identified by assessing the molecular similarity within compounds retrieved from the literature and DBH with a pairwise similarity. The fingerprint analyses showed that DBH has a structure similar to that of the most potent compounds acting as ligands for EcR, urease, and HIV-1 integrase recovered from the literature. Our molecular modeling experiments then indicate DBH is an excellent candidate for inhibiting the activity of EcR and urease. This interesting activity is added to the evident ability of DBH to complex ions, which, as already reported in the literature, enables its antimicrobial activity. The chelating nature of this compound could also be used to remove polluting metals as an ion detector and as a sensor. Furthermore, another attractive target, the HIV-1 integrase, was identified thanks to our docking studies. Further optimization of the structure of DBH could provide an excellent starting point for developing new inhibitors using this novel information. We used the already deposited .cif file as a starting point to perform a crystal morphology prediction. Overall, this study may help understand compound solubility, a thermodynamic property. It also provides information on a mainly kinetic-driven phenomenon such as dissolution rate. It sheds light on the crystal habit, a surface property, and helps the comprehension of mechanical properties, affecting API tableting [79]. We also predicted the possible effects of solvent on the real crystal growth, i.e., the real experimental conditions used for crystallization. All of this information could be of great help when considering potential issues with DBH tableting.

## Figures and Tables

**Table 1:** Predicted free energies of binding (Δ*G*, kcal/mol) and inhibition constant (*K*_i_, nM) for the EcR receptor.

Compound	Predicted Δ*G*	Predicted *K*_i_	Experimental *K*_i_
Ponasterone A ^a^	−11.37	4.6	4.8 ^b^
DBH	−9.01	249.9	—

^a^ Co-crystallized ligand. ^b^ From reference [17].

**Table 2:** Predicted free energies of binding (Δ*G*, kcal/mol) and inhibition constant (*K*_i_, μM) for the urease receptor.

Compound	Predicted Δ*G*	Predicted *K*_i_	Experimental *K*_i_
Thiourea ^a^	−5.55	85.06	21.00 ^b^
DBH	−7.06	6.71	—

^a^ Co-crystallized ligand. ^b^ From reference [42].

**Table 3:** Predicted free energies of binding (Δ*G*, kcal/mol) and inhibition constant (*K*_i_, nM) for the HIV integrase receptor.

Compound	Predicted Δ*G*	Predicted *K*_i_	Experimental *K*_i_
CHEMBL3259898 ^a^	−8.62	476.8	49.00 ^b^
DBH	−7.10	6,246.0	—

^a^ Co-crystallized ligand. ^b^ From reference [45].

**Table 4:** MI faces and morphology predictions for DBH calculated by BFDH (upper) and GM (lower) methods.

**MI**		**BDFH**		
***h k l***	**Multiplicity**	***d_hkl_* (Å)**		**% of Total Facet Area ^a^**
1 1 0	4	12.641		40.749
2 0 0	2	14.724		15.253
1 1 -1	4	15.437		36.027
1 1 1	4	19.059		7.462
0 0 2	2	23.746		0.509
**MI**		**GM**		
***h k l***	**Multiplicity**	***d_hkl_* (Å)**	***E*_att_ (kcal mol^−1^)**	**% of Total Facet Area ^a^**
1 1 0	4	7.911	−56.874	50.624
1 1 -1	4	6.478	−60.875	41.367
1 1 -2	4	4.087	−89.625	0.241
1 1 1	4	5.247	−89.670	6.083
2 0 0	2	6.792	−93.379	1.686

^a^ % of total facet area is calculated as 100 × (*hkl* facet area)/(total surface area).

## Data Availability

No new data were created.

## Supplementary Materials

The following supporting information can be downloaded at: https://www.mdpi.com/article/10.3390/ijms24021425/s1. Figure S1: Workflow of the procedure used for the molecular modeling studies; Figure S2: Ponasterone A within the EcR receptor; Figure S3: Thiourea within the urease receptor; Figure S4: CHEMBL3259898 within the HIV integrase receptor.
