# Supplementary material for: 1,2-Dibenzoylhydrazine as a Multi-Inhibitor Compound: A Morphological and Docking Study
Source: Int J Mol Sci. 2023 Jan 11;24(2):1425. doi: 10.3390/ijms24021425 (PMC9864281; doi:10.3390/ijms24021425)
Supplement: Supplementary file 1 [file ijms-24-01425-s001.zip › ijms-2109391-supplementary.pdf]

Supporting Information

1,2-Dibenzoylhydrazine as a multi-inhibitor compound: a morphological and docking study

Vincenzo Patamia [1], Giuseppe Floresta [1], Chiara Zagni [1], Venerando Pistarà [1], Francesco Punzo [1] and Antonio Rescifina [1,*]

[1] Department of Drug and Health Sciences, University of Catania, V.le A. Doria 6, 95125 Catania, Italy; vincenzo.patamia@unict.it (Vi.Pa.), giuseppe.floresta@unict.it (G.F.), chiara.zagni@unict.it (C.Z.), vpistara@unict.it (V.P.), fpunzo@unict.it (F.P.), arescifina@unict.it (A.R.)
* Correspondence.; email address: arescifina@unict.it (A.R.); Tel. +39 095 738 4245)

Table of contents

Figure S1. Workflow of the procedure used for the molecular modeling studies S3

Figure S2. Ponasterone A within the EcR receptor S3

Figure S3. Thiourea within the urease receptor. S4

Figure S4. CHEMBL3259898 within the HIV integrase receptor. S4

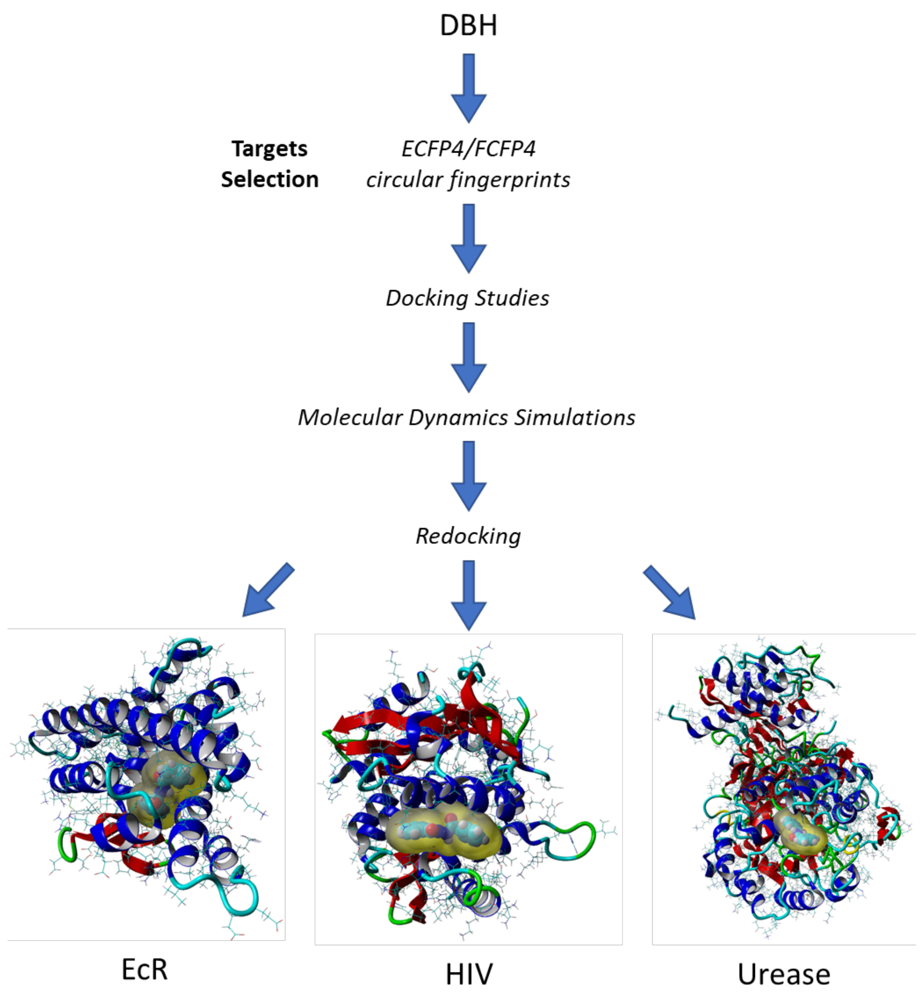

Figure S1. Workflow of the procedure used for the molecular modeling studies.

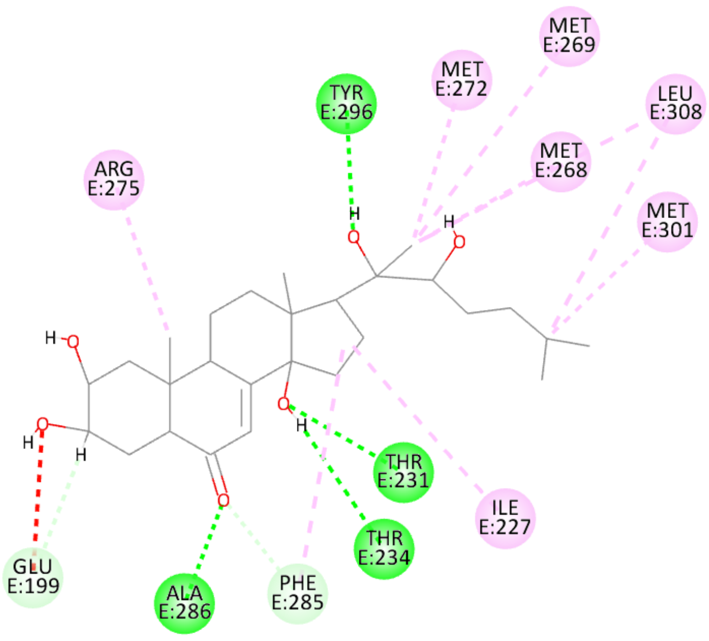

Figure S2. 2D picture of the Ponasterone A interactions within the EcR receptor.

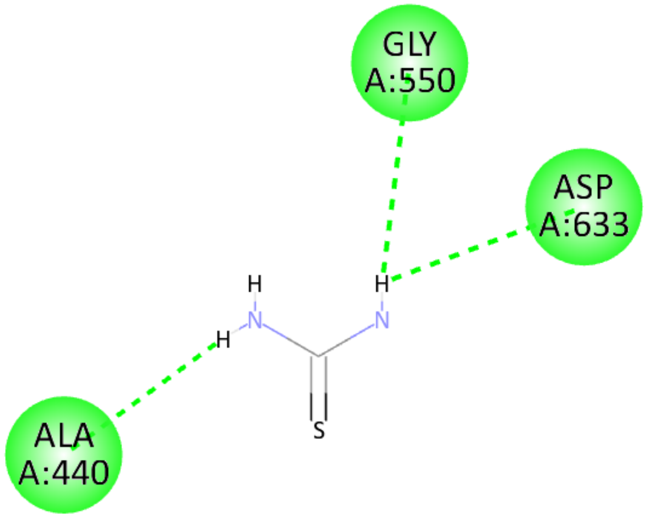

Figure S3. 2D picture of the Thiourea interactions within the urease receptor.

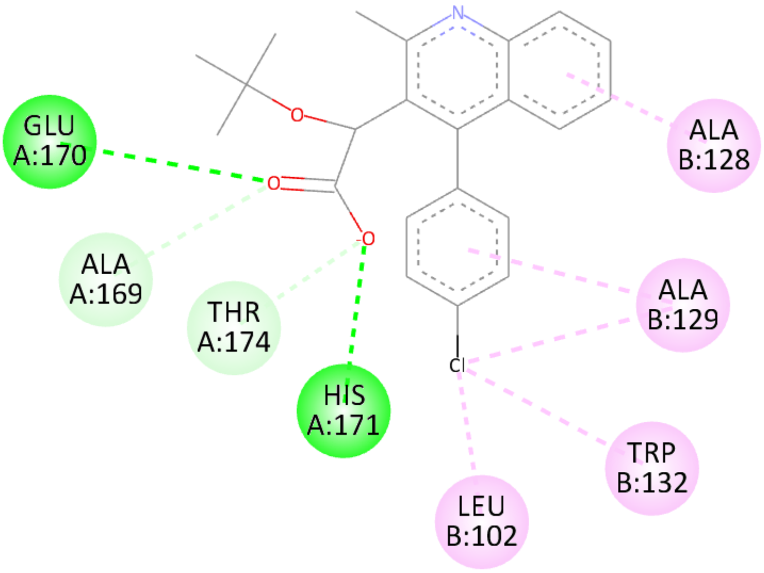

Figure S4. 2D picture of the CHEMBL3259898 interactions within the HIV integrase receptor.
